# The Making and Breaking of Social Ties During the Pandemic. Socio-Economic Position, Demographic Characteristics, and Changes in Social Networks

**DOI:** 10.3389/fsoc.2022.837968

**Published:** 2022-06-10

**Authors:** Ariane Bertogg, Sebastian Koos

**Affiliations:** ^1^Zukunftskolleg and Department of History, Sociology, Sports Sciences and Empirical Educational Sciences, University of Konstanz, Konstanz, Germany; ^2^Cluster of Excellence “The Politics of Inequality” and Department of History, Sociology, Sports Sciences and Empirical Educational Sciences, University of Konstanz, Konstanz, Germany

**Keywords:** COVID-19, social networks, social inequality, socio-demographic factors, social integration, social ties

## Abstract

Contact restrictions and distancing measures are among the most effective non-pharmaceutical measures to stop the spread of the SARS-CoV2 virus. Yet, research has only begun to understand the wider social consequences of these interventions. This study investigates how individuals' social networks have changed since the outbreak of the pandemic and how this is related to individuals' socio-economic positions and their socio-demographic characteristics. Based on a large quota sample of the German adult population, we investigate the loss and gain of strong and weak social ties during the pandemic. While about one third of respondents reported losing of contact with acquaintances, every fourth person has lost contact to a friend. Forming new social ties occurs less frequently. Only 10–15% report having made new acquaintances (15%) or friends (10%) during the pandemic. Overall, more than half of our respondents did not report any change, however. Changes in social networks are linked to both socio-demographic and socio-economic characteristics, such as age, gender, education, and migration background, providing key insights into a yet underexplored dimension of pandemic-related social inequality.

## Introduction

The COVID-19 pandemic and ensuing containment measures have had far-reaching implications for the lives of many people around the globe, affecting their health, incomes, and wellbeing. While much research has been devoted to understanding these ramifications, relatively little work has focused on the consequences of the pandemic for social networks, the relationships that define and are the essence of peoples' social lives. The beneficial nature of social ties, often called social capital, has been shown to be of crucial importance for economic, educational and health outcomes (see also Granovetter, 1974; Ferlander, 2007; Frank et al., 2008). Yet, during the pandemic social ties have been mainly understood as channels of contagion (Block et al., 2020; Karaivanov, 2020), and less as channels of support or resilience (Rashid and McGrath, 2020). The COVID-19 containment measures have imposed severe restrictions on in-person contacts and meeting opportunities, possibly threatening the continuation of old and the formation of new ties. While we know that contact restrictions have had detrimental effects on individuals' mental health (Gersons et al., 2020; Kuhn et al., 2020), we know surprisingly little about their potential longer-term effects on peoples' social networks (an exception being: Arpino et al., 2020).

It is important to understand the impact of the pandemic on social networks for a number of reasons: First, under normal circumstances, network changes occur slowly (Wrzus et al., 2013; Fischer and Beresford, 2015). A global pandemic constitutes an exogenous—unforeseen and irreversible—shock and may have sped up network developments. Second, sudden external shocks, such as societal crises, may weaken social ties (Rivera et al., 2010; Hilmar, 2020). Negative network changes may thus undermine social cohesion and limit the capabilities for individual and societal recovery. Third, social network resources are unequally distributed, favoring those in higher socio-economic positions. Individuals with more resources may have been better able to absorb network shocks and prevent a loss of social ties. A number of studies warn that the pandemic may have reinforced existing social inequalities (Ohlbrecht and Jellen, 2020; Engzell et al., 2021; Gauthier et al., 2021). Changes in social networks may be another—yet under-researched—mechanism of exacerbating social inequality, wellbeing and access to resources. This should be particularly true if individuals who already belong to vulnerable groups—are more strongly affected by a loss of network ties.

In this study we thus ask: How have social networks of individuals changed since the outbreak of the COVID-19 pandemic? Who has gained or lost friends or acquaintances? Do these changes vary between individuals with different socio-demographic and socio-economic characteristics?

To answer these questions we use four measures which were specifically designed for this purpose and were fielded in the context of a large topical, multi-purpose, online survey in early May 2021 in Germany. In distinguishing between acquaintances (persons one meets rather regularly, but does not know well) and friends (people one knows well and likes a lot), we can not only provide a rather fine grained-analysis of quantitative network changes during the pandemic, but also gain some insight into the development of so-called weak and strong ties over the course of the pandemic. The timing of the survey, ~14 months after the first lockdown measures had been implemented, allows assessing the longer-term pandemic-related network changes for the first time. In Germany, a first, strict lockdown was imposed between March and May 2020, a second, lenient one in fall 2020, and a third, again strict, one between December 2020 and May 2021 (Hale et al., 2021). During the first and the third lockdown, schools remained closed, home office was mandatory where possible, and most sports, cultural, and leisure facilities remained closed. Moreover, private gatherings of more than five people were placed under a ban. Thus, social life came to a halt, restricting the opportunities for both meeting existing social ties and forming new ties. By collecting our data just at the end of this second long and strict lockdown, we are likely to observe changes due to these lockdowns before social relationships may have recovered again.

These contact restrictions are likely to have implications for the number of people in one's social network. On the one hand, people might have lost ties (“breaking”) (see Borkowska and Laurence, 2020). On the other hand, there were also potentials for positive changes, such as the gain of new ties (“making”). One can thus expect to observe four types of network changes due to the pandemic: First, we assume that a loss of network ties has been common due to contact restrictions and limited opportunities to meet. Moreover, the situational factors which normally enable friendship formation (Fehr, 2008) have been limited by the containment measures, suggesting that friendship formation has been difficult during the crisis. Second, however, given the wave of helping arrangements which emerged during the first lockdown (Carlsen et al., 2020), and the temporarily relaxed restrictions during summer 2020, new ties might have been gained. Third, one could assume that both—gain and loss—may have occurred within the network of the same individual. Finally, some people may have very stable networks which were not affected by the lockdown measures.

Individuals from different socio-demographic and socio-economic groups might have different risks for these network changes. First, people in better socio-economic positions, i.e., higher education and/or income, usually have larger social networks and more resources to maintain these networks (Pichler and Wallace, 2007). On the one hand, this potentially allows cultivating social ties—also at a distance. On the other hand, they also more often worked from home during the pandemic—which suggests that they might have lost contact with colleagues. Employment not only generates resources, but also access to social network, thus, those employed might be better able to maintain their social networks. Second, specific socio-demographic risk factors might affect changes in social networks. Age is a pivotal factor: Social ties usually become more stable as people age (Wrzus et al., 2013). Women more often than men were socialized to feel responsible for organizing a couple's or a family's social ties and they are more likely to engage in pro-social behaviors (Hochschild, 1979; Rossi and Rossi, 1990). Thus, their social network ties might be more vulnerable to a the changed opportunities for socializing. Not least having a migration background may further restrict opportunities of cultivating social ties, e.g., through a language barrier, or because close social ties live far away. On the other hand, diaspora networks are often densely knit and provide comprehensive informal exchange.

## Methods

Our analyses are based on a multi-purpose online survey “Living in exceptional circumstances”^1^ Wave 3 (spring 2021) implemented into the Respondi online access panel. It draws on a quota sample of the German adult population, based on region, gender, age, and educational level. Compared to German census data, our sample is broadly comparable. Lower educated respondents are underrepresented (see Supplementary Table 1) and people form East Germany were deliberately oversampled therefore we use population-based weights throughout our analyses.

Three waves of data were collected during three different stages of the pandemic. In this study, we rely on data collected during the third panel wave, in early May 2021. This period captures the end of a long and strict, third, lockdown, which had started before Christmas. The end of the lockdown varied regionally and was tied to the averaged 3-day incidence level of COVID-19 infections in each county, according to a new law, the so-called “federal emergency brake” (Bundesnotbremse). The third panel wave was selected, because only in that wave was it feasible to ask for longer-term network changes due to the pandemic. Overall, we observe 4,027 individuals aged between 18 and 91 years in the third wave, after listwise deletion of cases with missing values, our analytical sample comprises 3,378 persons.

### Dependent Variables

The phenomenon under study in this article are changes in individuals' social networks. We measure four types of change in networks during the pandemic, reflecting two dimensions of positive (“making”), respectively, negative (“breaking”) change and change of strong (friends), respectively, weak (acquaintances) ties. They were measured with four self-reported dichotomous items (see Table 1).

**Table 1:** Types of network changes.

**Item**	**Gain**	**Loss**
*Positive Change (“Making”)*		
I have gained new acquaintances during the pandemic	✓	
I have gained new friends during the pandemic	✓	
*Negative Change (“Breaking”)*		
I have lost friends during the pandemic		✓
I have lost acquaintances during the pandemic		✓

### Socio-Demographic and Socio-Economic Factors

Risks for network changes arise from both socio-demographic and socio-economic variables. Socio-demographic factors span individual's gender, age, and immigration history. Gender was measured with a dichotomous variables (1 = Female). Age was measured in three broader groups in order to allow for non-linear effects (18–35, 36–59, 60 years, or older). Germany is ethnically less diverse than many other Western countries, but has a large share of first and second generation migrants (respondent or both parents born abroad), which is another social stratification dimension. We thus create a dichotomous variable indicating whether the respondent him- or herself was born in another country than Germany, or whether both his parents were born outside of Germany (1 = Yes). Socio-economic factors include educational level, income and employment status. Education was measured using information on the highest level of professional education attained, and was recoded into three categories: Low (At most compulsory schooling), Intermediate (A-Levels or Vocational Training), and High (Tertiary Education). We have information on the disposable household income, which was recoded into six categories (<900 Euros, 900–1,499 Euros, 1,500–1,599 Euros, 2,600–3,999 Euros, 4,000–5,999 Euros, 6,000 Euros or more). Employment situation was measured with three categories: Employed, Retired and Economically inactive (the latter including those who are homemaking, permanently ill or disabled, in education, or unemployed).

### Additional Control Variables

Finally, we also adjust our models for a number of additional control variables, which could potentially contribute to network change. First of all the regional characteristics may play a role, e.g., with their infrastructure for meeting other (or new) people. Thus, we control for whether our respondents live in an urban or rural area (1 = Urban), and whether they were born in the Eastern part of Germany (former GDR). Further controls include individuals' own COVID-19 health risk profiles, measured with four variables: We control for self-rated health in general (measured on a five-point scale from very bad to very good, whether one belongs to a risk group for severe COVID-19 pathologies (1 = Yes), whether one has been infected with COVID-19 (1 = Yes) or knows someone who has been infected (1 = Yes). Third, potential changes in networks might depend on pre-pandemic network characteristics. We measured them with two summative index variables, including both the frequency of contact and the number of contacts with strong (family, friends) and weak ties (colleagues, people from voluntary associations and churches, other acquaintances). For a total of six groups of potential network partners, we asked for the frequency of contact and in-person meetings before the pandemic (ranging from 1 to 5, from never to almost daily), and summed them up so that higher values indicate more frequent contact with a larger number of different ties. Fourth, life course situations and transitions might play a role. We adjust for whether one lives with a partner or spouse and has children in the household or outside the household. Finally, we asked for seven life course transitions in the domain of education, work, partnership and housing. In addition, we also control for whether he or she worked from home.

### Analytical Strategy and Presentation of the Results

A first descriptive analysis is followed by two sets of multivariate analysis of underlying forces that drive network changes. All analyses apply population-based weights. First, we are interested in the four different types of changes separately. Since these were measured as yes-no questions, we apply logistic regression models. Being mainly interested in the effects of socio-demographic (such as gender, age, migration status) and socio-economic factors (such as education, income, and employment status), we estimate three sets of models. The first model only includes each of these explanatory variables separately (bivariate or “raw” effects). The second model estimates the effects of all these six variables simultaneously. Finally, in the third model, we adjust for the control variables (described above) as they may be confounders (e.g., West Germans having higher incomes) or explain parts of the effects of our six core variables (e.g., employment may be more protective of networks if not resuming to working remotely). The results from the first (bivariate) and second models (all socio-demographic and socio-economic variables) are presented in **Figure 2**. The regression tables, containing all models including the ones with additional controls can be found in Supplementary Table 2.

Second, we are also interested in whether positive and negative changes may co-occur, e.g., someone both losing and gaining ties. For that reason, we collapsed all gains (i.e., either gain of acquaintances, friends, or both) and losses (i.e., either loss of acquaintances, friends, or both) into two dichotomous variables indicating whether one had gained, respectively, lost some ties (1 = yes), independent of whether these were friends or acquaintances. From these variables, we construct a categorical variables indicating whether (1) respondents reported no change at all, (2) respondents reported only gain of ties, (3) respondents reported both gain and loss of ties, and (4) respondents reported only loss of ties. This categorical variable is analyzed using a multinomial logistic regression. The result from this model is presented in **Figure 3**, and the respective regression results can be found in Supplementary Table 3.

In **Figures 2**, **3** the results are presented as Average Marginal Effects, which can be interpreted as percentage changes in the likelihood of experiencing a particular network change.

## Results

### Changes in Network: Frequency and Co-occurrence

**Figure 1:**
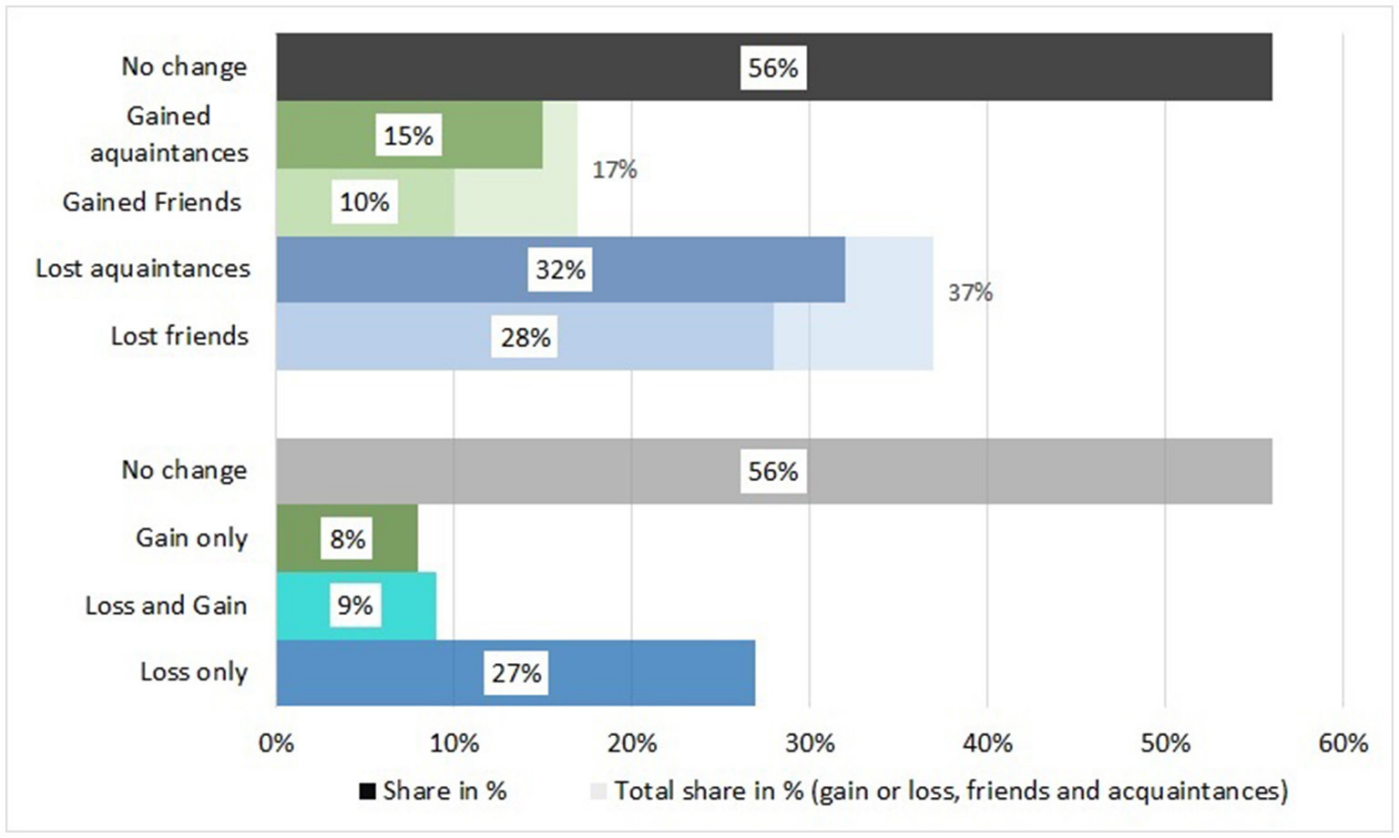
Change in social ties. Source: Survey “Living in exceptional circumstances,” Wave 3 (spring 2021). *n* = 3,713 respondents, 18–98 years. Own calculations, applying population weights based on gender, age, education, migration background and region, calculated on the basis of German census data.

Our descriptive analysis of the different types of changes in social networks (Figure 1) shows that more than one third of all respondents (37%) report losing ties during the pandemic, while only 17% report gaining new ones. Among those who lose ties, the majority loses both acquaintances and friends. About one in three loses an acquaintance, and about one in four loses a friend. Among those who gain new ties, 15% gain an acquaintance and 10% gain a friend. Nevertheless, a slight majority of respondents does not report any change in their social relationships (55%). As regards co-occurring changes, we find that more people (27%) report only loss than people who report only gains (8%). Less than one in 10 reports both loss and gain (9%). Thus, overall, loss is the dominant trend, and only a small group can compensate this through gain of new ties.

### Socio-Demographic and Socio-Economic-Disparities

**Figure 2:**
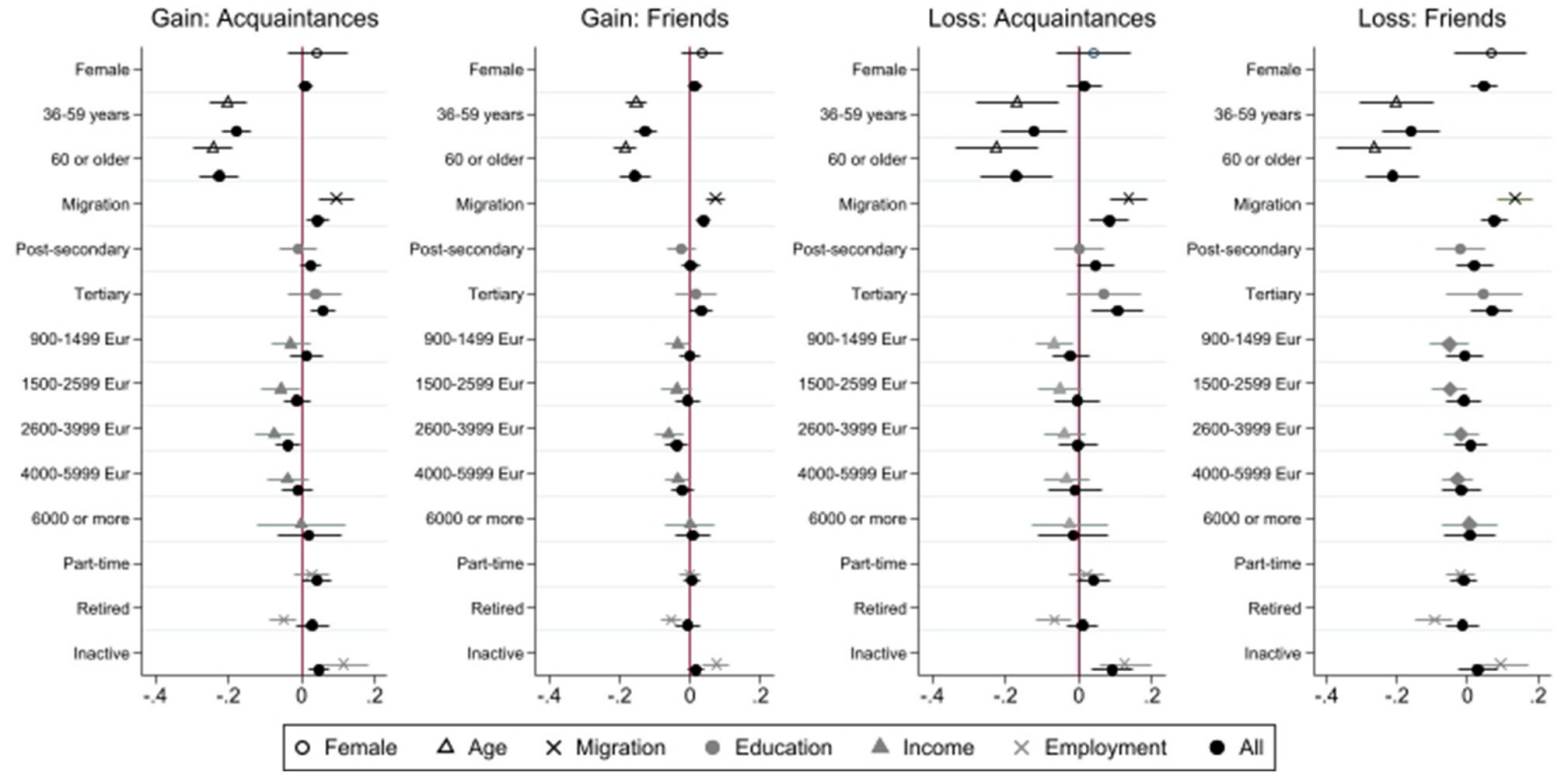
Socio-Demographic and Socio-Economic Determinants of Network Change. Source: Survey “Living in exceptional circumstances,” Wave 3 (spring 2021). *n* = 3,713 respondents, 18–98 years. Multivariate logistic regression models, weighted, Average Marginal Effects (difference in likelihood in percent). Bivariate coefficients for each socio-demographic resp. socio-economic variable (coefficients: “Female”, “Age”, “Migration”, “Education”, “Income”, and “Employment”) and coefficients from simultaneous model estimation, including controls for urbanity and East Germany (coefficient “All”). Reference categories: Man, Age 16–34, No Migration Background, Compulsory Education, Income <900 Euros/month, Full-time employment. For full set of coefficients, see in Supplementary Table 2. For comparison with full model (including additional controls), see Supplementary Table 2 (last column).

In the next step, we present the multivariate findings for socio-demographic and socio-economic differences in the four types of change (Figure 2). These coefficients were obtained including all socio-demographic and socio-economic variables simultaneously (but not adjusting for additional controls).

Starting with socio-demographic factors, women are more likely to report loss of friends than men (which is robust when adjusting for control variables), but not differ statistically significantly from men for other types of network changes. Older age is associated with lower likelihood of network changes, which applies to gain and loss for both acquaintances and friends. Moreover, these findings are robust when adjusting for controls. Migrants have a higher likelihood of all four types of network changes, both losing and making friends and acquaintances. This is robust when adding control variables, except for finding new acquaintances. For this group, the effects of losing ties is however somewhat stronger, compared to gaining new ties. Overall, socio-demographic characteristics are quite strongly related to network changes during the pandemic.

Regarding socio-economic factors, we find that those with tertiary education are more likely to gain acquaintances, as well as lose friends and acquaintances than those with compulsory education. Those with intermediate education (e.g., A-Levels or Vocational Training) are also more likely to lose acquaintances, but not friends. These effects are only statistically significant, when controlling for socio-demographic and other socio-economic variables and vanish in the bivariate and the full model. Thus, there seems to be no robust effect of education level on changes in social networks. Similarly, income is not systematically correlated with gain or loss of ties. The group of middle class earners (2,600–3,000 Euros) is slightly less likely to gain ties, but this effect does not hold when adjusting for controls (see Supplementary Table 2). Finally, with regard to employment status, we find that economically inactive persons (such as students) are more likely to experience both gain and loss of acquaintances and friends, than full-time employed respondents. People that are retired have a lower likelihood of all four types of network changes in the bivariate models. The effects however vanish, when controlling for age (model 2). Thus, people that are active in the labor market seem to have more stable networks, than people that are inactive, but not than people who are retired. In sum, the effect of socio-economic variables on changes in social networks during the pandemic is rather limited. Only employment status is related to different types of network changes.

- Moreover, the additional controls exhibit some interesting effects themselves (for all results, including full models, see Supplementary Table 2). Interestingly people residing in East Germany have a lower likelihood of losing friends and acquaintances. Both pre-pandemic networks and individual COVID-19 risk profiles structure the opportunities for network changes during the pandemic. We find that more frequent contacts with different types of weak ties increase the likelihood of gaining friends and acquaintances, as well as losing acquaintances, and more frequent contacts with strong ties promote the formation of new ties with acquaintances during the pandemic. With regard to health risks, we find that respondents' poor health increases the likelihood of loss of both acquaintances and friends.

### Co-occurrence of Network Changes?

**Figure 3:**
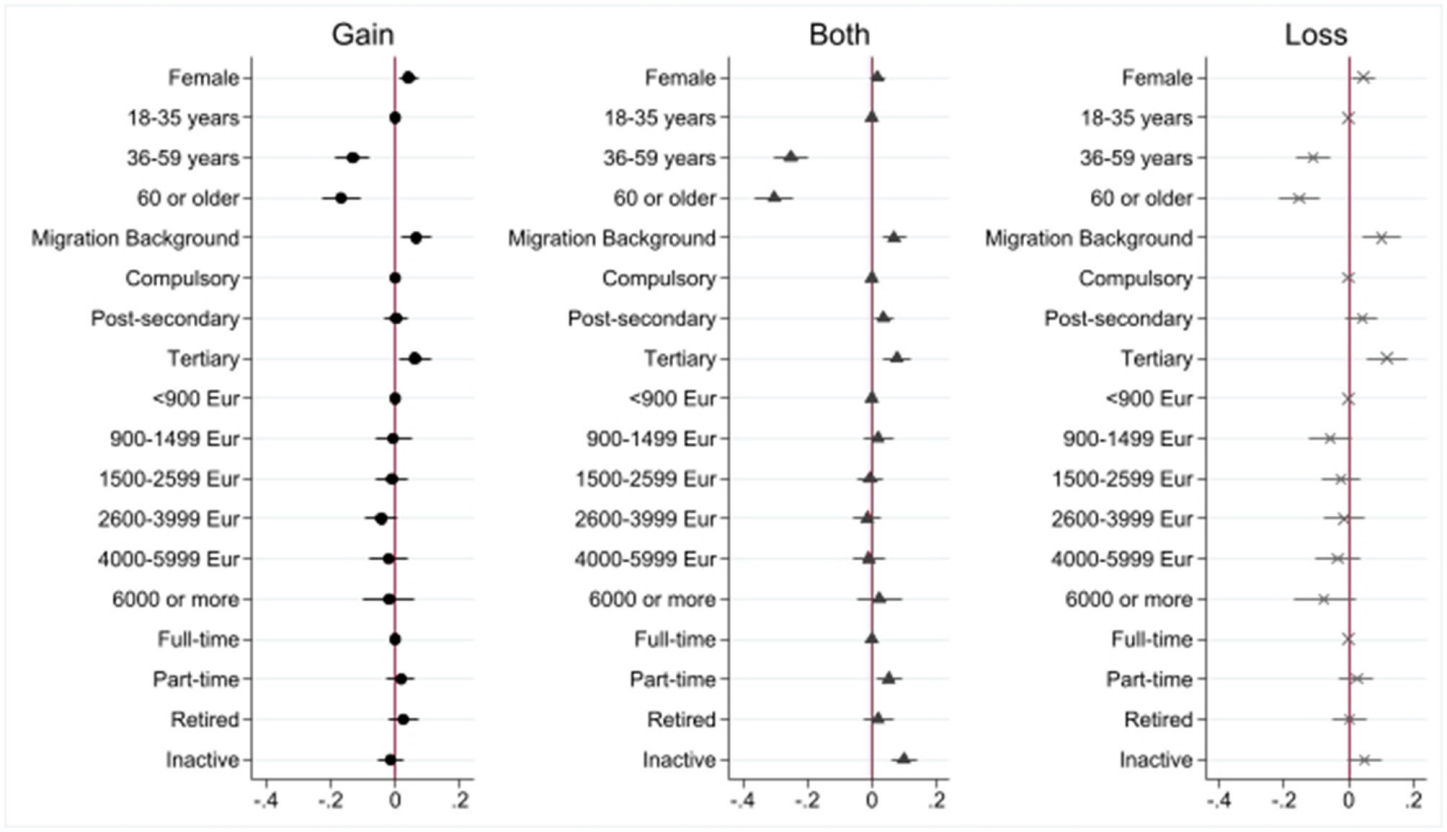
Co-occurring Changes. Source: Survey “Living in exceptional circumstances” Wave 3 (spring 2021). *n* = 3,713 respondents, 18–98 years. Multivariate logistic regression models, weighted, Average Marginal Effects (difference in likelihood in percent). Gain resp. loss of friends and acquaintances jointly modeled (1 = gain/loss of either). For coefficients, see Supplementary Table 3.

Finally, we ask whether gains and losses may also co-occur, potentially offsetting some of the negative or positive effects of new tie formation or loss of social capital. Again, we are most strongly interested in the disparities between individuals with different socio-demographic and socio-economic characteristics. Figure 3 shows the Average Marginal Effects from the multinomial logistic regression model including all socio-demographic and socio-economic variables (simultaneous estimation), but not additional controls. For the detailed results, please refer to Supplementary Table 3. The coefficients are to be interpreted in relation to the reference category, “No change.” Combining tie changes of both acquaintances and friends, the results are not full comparable to the above models and show a slightly different picture of the overall changes in network ties. Compared to males, females are more likely to report either gain or loss of contacts than men compared to reporting no change. Again, older people are less likely to report network changes than younger people. Moreover, migrants are more likely to report all types of changes than non-migrants. Turning to the socio-economic variables, we find that people with tertiary education have a higher likelihood to report all types of changes than people with lower (at least compulsory) education. While, again, income has no statistically discernible effect, inactive respondents have a higher likelihood to report both changes at the same time, compared to people in full time employment. This also applies to people employed part-time. Thus, the socio demographic variables show robust relationships to network changes. In these more encompassing models, that do not differentiate between friends and acquaintances higher education is also related to all types of network changes.

## Discussion

The networks of many people have changed considerably during the COVID-19 pandemic, and—as expected—the contact restrictions have taken their toll: Within only 1 year, almost one in two has experienced a change in their networks. More than one in three has experienced a shrinkage of their networks, and most people who lost ties lost both acquaintances and friends. At the same time, losses of social ties have hardly been compensated by gaining new ties. However, we also observe positive changes. Despite the aggravating conditions during the various lockdowns in Germany, about one in six has gained new ties. In sum, compared to evidence on network shrinkage in non-pandemic times, the documented changes for the analyzed time-span of 14 months since the outbreak of the pandemic until the survey took place, are considerable (Wrzus et al., 2013).

Yet, unfortunately, there is exists no pre-pandemic study of network changes in the general population that would allow a direct comparison of network changes, as measured in our study. However, in another wave of our survey fielded in April/May 2022, we asked our respondents repeatedly to indicate the types of network changes experienced, and we also asked how network changes compared to pre-pandemic times (more or less frequently, about the same). First analyses on raw, unweighted data from this fourth wave show that after 2 years of the pandemic, between 26 and 33 per cent report having lost ties, while between 15 and 19% report having gained ties. While the figures of loss are very comparable to the figures presented in this article based on data fielded 1 year earlier, the figures for gain in ties are higher. With regard to the subjective assessment whether change of ties had become more or less common, more than two thirds of those who report a loss of ties, indicate that this happened more frequently than before the pandemic. Among the fewer people who gained new ties, only about 40% report that this happened more frequently than before the pandemic. This suggests that the diminishing effects of the pandemic on social networks have been substantial, exceeding “normal” changes considerably.

Network changes may have longer-term consequences for social inequality, as strong and weak social ties are important for wellbeing, receiving support, coping with crises, but also finding a job and reconciling work and family. This is particularly critical if social network changes occur unequally across social groups, and negative changes predominantly affect those who were already in a vulnerable position before the pandemic. Thus, we analyzed how these network changes differ between socio-demographic and socio-economic groups. With respect to socio-demographics, we found that women are more likely than men to lose friends, and the multinomial models, using an undifferentiated measure of the type of tie, shows that females are more likely to report all types of network changes, including making new contacts. Some literature suggests that this might be due to women being socialized to be “kinkeepers,” and typically, they are in charge for organizing close social ties (Rossi and Rossi, 1990). During the pandemic, our first results suggest, it is rather their close, but not their weak ties that were more prone to change than men's.

Young adults generally have more volatile networks and experience more change. Old age protects from loss of ties, but also decreases the likelihood of making new ties. This is in line with previous research according to which networks stabilize as people get older and close network ties are becoming more important, whereas making new ties becomes less important (Carstensen, 1992). Conversely, networks are more volatile among young adults than in mid-adulthood, not least because typically young people experience a higher density of life course transitions in the domains of education, employment, and partnership (Arnett, 2000). Individuals with a migration background have a higher likelihood of experiencing loss and gain of both types of social ties. Thus, migrants seem to have more volatile social networks during the pandemic. For this group the likelihood of losing contacts to friends and acquaintances is somewhat stronger than for finding new ones. Thus, the potential social disintegration of migrants seems to have been aggravated by the pandemic. This has also potential negative implications for migrants' ability to cope with crisis induced hardship compared to non-migrants.

Finally, more highly educated people have more volatile networks in the pandemic compared to people with lower levels of education. The likelihood of losing contacts is higher than of gaining ties in this group, suggesting that higher education is not a buffer against social network changes. Income is not systematically related to network changes during the pandemic. While it is often claimed that the more affluent people have more ties via membership in clubs, leisure and voluntary organizations, these facilities were mostly closed due to the containment measures and therefore restrict the opportunities for cultivating social ties in this group. We found that the retired are better protected from network changes than people being active in the labor market, while the inactive (such as students) have a higher chance for losing and gaining ties. In sum, rather than socio-economic status (SES) variables, it is socio-demographic variables that are robustly related to network changes during the pandemic.

These findings with regard to socio-demographic differences in network changes imply that the pandemic has not changed social networks more in economically less privileged groups. Considering the effect of tertiary education, we rather find the opposite. Yet, apart from this, the young as well as migrant groups suffer the most network changes, specifically higher levels of losing ties. Our results also suggest the need for future research into the pandemic-related mechanisms why some socio-demographic groups, such as women, the young and migrants, are more likely to lose ties—other than their generally higher network volatility. With regard to gender differences, a number of studies has documented a larger increase in housework and childcare, as well as work-family-stress among women than among men (Kulik and Liberman, 2013; Kuhn et al., 2020). Women more often suffered from a time squeeze than men, which could be an explanation for their limited ability to keep in touch with friends. With regard to age differences, network volatility associated with young adulthood (Arnett, 2000) may have increased due to the difficulty of finding new partnerships or jobs. Future research will be needed to better understand these risks, and to see whether these groups were able to catch up with the forgone opportunities for maintaining old ties and forming new ones after the pandemic.

Given the warnings against a second, mental health, pandemic, and the threatening increasing inequalities, understanding the roots and the long-term consequences of various types of network changes due to the COVID-19 crisis is a pivotal task for research. Our study contributes to this field with a first, systematic investigation into network changes. Our findings suggest that the dissolution of ties may be problematic in the longer run because changes in networks act as a mediating mechanism for wellbeing during the COVID-19 crisis. Social networks are an important buffer against the adverse consequences of crises, and a crucial source of social resilience (Hurlbert et al., 2017; Carlsen et al., 2020). Thus, the chances for recovering from the pandemic ultimately depend on a society's social capital, which the pandemic threatens to undermine. The long-term psychological, social, and economic consequences of broken ties remain to be addressed by future research.

## Nomenclature

### Resource Identification Initiative

To take part in the Resource Identification Initiative, please use the corresponding catalogue number and RRID in your current manuscript. For more information about the project and for steps on how to search for an RRID, please click here.

## Data Availability Statement

The data used for this article are under embargo and will be released *via* Gesis thereafter. For access to a reduced replication data set, please contact the authors directly.

## Author Contributions

All authors listed have made a substantial, direct, and intellectual contribution to the work and approved it for publication.

## Funding

This study was supported by German Research Foundation (DFG) EXC-2035/1 – 390681379 (to SK).

## Conflict of Interest

The authors declare that the research was conducted in the absence of any commercial or financial relationships that could be construed as a potential conflict of interest.

## Publisher's Note

All claims expressed in this article are solely those of the authors and do not necessarily represent those of their affiliated organizations, or those of the publisher, the editors and the reviewers. Any product that may be evaluated in this article, or claim that may be made by its manufacturer, is not guaranteed or endorsed by the publisher.

## Footnotes

^1^For documentation of the survey program, please refer to https://www.exc.uni-konstanz.de/en/inequality/research/covid-19-and-inequality-surveys-program/documentation/.
