# Supplementary material for: The Making and Breaking of Social Ties During the Pandemic. Socio-Economic Position, Demographic Characteristics, and Changes in Social Networks
Source: Front Sociol. 2022 Jun 10;7:837968. doi: 10.3389/fsoc.2022.837968 (PMC9226385; doi:10.3389/fsoc.2022.837968)
Supplement: Supplementary file 1 [file Data_Sheet_1.docx]

Supplementary Material

The Making and Breaking of Social Ties during the Pandemic. Socio-Economic Position, Demographic Characteristics, and Changes in Social Networks

|  | **Sample (unweighted)** | **Population (census)** |
| --- | --- | --- |
| Highest educational level attained: |  |  |
| Lower education | 12.8 % | 35.8 % |
| Secondary schooling | 65.2 % | 30.5 % |
| Higher education | 21.9% | 33.6 % |
| Female | 48.4 % | 50.1 % |
| Age: |  |  |
| 18-39 years | 21.9 % | 31.6 % |
| 40-59 years | 44.3 % | 35.2 % |
| 60+ years | 33.7 % | 33.2 % |
| East | 27.6 % | 19.7 % |

**Supplementary Table 1: Comparing unweighted sample to population census data.**

Source: Survey "Living in exceptional circumstances", Wave 3 (spring 2021). n= 3,713 respondents, 18-98 years. Note: East Germany was deliberately oversampled in the survey.

|  | (1) | (2) | (3) | (4) | (5) | (6) | (7) | (8) | (9) | (10) | (11) | (12) |
| --- | --- | --- | --- | --- | --- | --- | --- | --- | --- | --- | --- | --- |
| Dependent Variable | **Gain** |  |  |  |  |  | **Loss** |  |  |  |  |  |
| Gain / Loss of… | Acquaintances | | | Friends | | | Acquaintances | | | Friends | | |
| Model | Bi-variate | Reduced | Full | Bi-variate | Reduced | Full | Bi-variate | Reduced | Full | Bi-variate | Reduced | Full |
| Woman | 0.043 | 0.010 | 0.011 | 0.035 | 0.013 | 0.018 | -0.041 | 0.015 | 0.013 | -0.041 | 0.047^**^ | 0.044^*^ |
| Age: 18-35 years (ref.) | |  |  |  |  |  |  |  |  |  |  |  |
| 36-59 years | -0.243^***^ | -0.178^***^ | -0.083^***^ | -0.153^***^ | -0.127^***^ | -0.055^***^ | -0.169^**^ | -0.122^***^ | -0.102^***^ | -0.087 | -0.160^***^ | -0.125^**^ |
| 60 or older | 0.096^***^ | -0.225^***^ | -0.123^***^ | -0.185^***^ | -0.156^***^ | -0.078^***^ | -0.226^***^ | -0.171^***^ | -0.132^***^ | -0.125^*^ | -0.212^***^ | -0.167^***^ |
| Migration background | 0.096^***^ | 0.044^*^ | 0.031 | 0.074^***^ | 0.038^**^ | 0.029^*^ | 0.137^***^ | 0.083^**^ | 0.066^*^ | 0.134^***^ | 0.077^***^ | 0.060^**^ |
| Highest educational level attained: Compulsory (ref.) |  |  |  |  |  |  |  |  |  |  |  |  |
| Post-secondary | -0.009 | 0.026 | 0.015 | -0.026 | 0.002 | -0.002 | 0.002 | 0.045^*^ | 0.030 | -0.020 | 0.019 | 0.006 |
| Tertiary | 0.038 | 0.059^**^ | 0.035 | 0.016 | 0.032 | 0.026 | 0.068 | 0.106^***^ | 0.072 | 0.046 | 0.070^**^ | 0.041 |
| Income: <900 Eur/month (ref.) | |  |  |  |  |  |  |  |  |  |  |  |
| 900-1499 Eur | -0.030 | 0.013 | 0.008 | -0.035^*^ | -0.001 | -0.004 | -0.068^**^ | -0.023 | -0.023 | -0.052 | -0.007 | -0.009 |
| 1500-2599 Eur | -0.057^*^ | -0.012 | -0.001 | -0.037 | -0.007 | 0.005 | -0.052 | -0.004 | 0.012 | -0.049 | -0.010 | 0.008 |
| 2600-3999 Eur | -0.074^**^ | -0.037 | -0.016 | -0.060^**^ | -0.038^*^ | -0.014 | -0.040 | -0.003 | 0.013 | -0.018 | 0.009 | 0.031 |
| 4000-5999 Eur | -0.037 | -0.010 | 0.001 | -0.035 | -0.022 | -0.006 | -0.033 | -0.011 | 0.006 | -0.029 | -0.017 | 0.010 |
| 6000 or more | -0.001 | 0.020 | 0.028 | 0.001 | 0.002 | 0.017 | -0.025 | -0.014 | -0.002 | 0.006 | 0.007 | 0.029 |
| Full-time (ref.) |  |  |  |  |  |  |  |  |  |  |  |  |
| Part-time | 0.028 | 0.043^*^ | 0.039^*^ | -0.000 | 0.006 | 0.007 | 0.020 | 0.040 | 0.030 | -0.019 | -0.011 | -0.020 |
| Retired | -0.050^*^ | 0.029 | 0.055^*^ | -0.053^***^ | -0.007 | 0.013 | -0.068^**^ | 0.010 | 0.012 | -0.094^***^ | -0.014 | -0.017 |
| Inactive | 0.115^**^ | 0.048^**^ | 0.057^***^ | 0.074^***^ | 0.016 | 0.025^*^ | 0.125^***^ | 0.091^***^ | 0.093^**^ | 0.094^*^ | 0.029 | 0.023 |
| Urban |  | 0.013 | 0.009 |  | -0.008 | -0.011 |  | 0.012 | 0.010 |  | 0.006 | 0.003 |
| East |  | 0.019 | 0.019 |  | 0.014 | 0.012 |  | -0.038^*^ | -0.034 |  | -0.038^*^ | -0.032^*^ |
| Pre-pandemic networks: strong ties | | | 0.019^**^ |  |  | 0.008 |  |  | 0.009 |  |  | 0.001 |
| Pre-pandemic networks: weak ties | | | 0.031^***^ |  |  | 0.030^***^ |  |  | 0.025^*^ |  |  | 0.019 |
| Health |  |  | 0.001 |  |  | 0.004 |  |  | -0.041^***^ |  |  | -0.038^***^ |
| Belongs to risk group |  |  | 0.015 |  |  | 0.000 |  |  | -0.015 |  |  | -0.010 |
| Has been infected |  |  | -0.009 |  |  | 0.033 |  |  | 0.032 |  |  | 0.021 |
| Someone in network was infected |  |  | 0.045^**^ |  |  | 0.007 |  |  | 0.086^***^ |  |  | 0.063^***^ |
| Partnered |  |  | -0.035^*^ |  |  | -0.021 |  |  | -0.037 |  |  | -0.033 |
| No children (ref.) |  |  |  |  |  |  |  |  |  |  |  |  |
| Minor children in household |  |  | -0.014 |  |  | -0.002 |  |  | -0.006 |  |  | 0.002 |
| Not in household/Adult |  |  | -0.033 |  |  | -0.021 |  |  | 0.018 |  |  | 0.013 |
| Works from home |  |  | 0.001 |  |  | -0.014 |  |  | 0.038 |  |  | 0.024 |
| Started new education |  |  | 0.015 |  |  | 0.013 |  |  | 0.026 |  |  | 0.037 |
| Stopped / Finished education |  |  | -0.009 |  |  | -0.010 |  |  | -0.017 |  |  | 0.010 |
| Started (new) job |  |  | 0.045^**^ |  |  | 0.001 |  |  | 0.088^*^ |  |  | 0.070^**^ |
| Quit or lost a job |  |  | 0.014 |  |  | 0.019 |  |  | 0.064 |  |  | 0.085^**^ |
| Started a new relationship |  |  | 0.107^***^ |  |  | 0.114^***^ |  |  | -0.022 |  |  | -0.051 |
| Ended a relationship |  |  | 0.015 |  |  | 0.007 |  |  | 0.072^*^ |  |  | 0.077^*^ |
| Moved |  |  | 0.028 |  |  | 0.019 |  |  | 0.029 |  |  | 0.042 |
| Observations | 3713 | 3713 | 3713 | 3713 | 3713 | 3713 | 3713 | 3713 | 3713 | 3713 | 3713 | 3713 |

**Supplementary Table 2: Bivariate Effects, Reduced, and Full Models.** Source: Survey "Living in exceptional circumstances", Wave 3 (spring 2021). n=3713 respondents, 18-98 years. Multivariate logistic regression models, weighted, Average Marginal Effects (difference in likelihood in percent). Bivariate: Separate models for each socio-economic and socio-demographic predictor calculated (displayed in the same column). Reduced Model: Only socio-demographic and socio-economic-factors. Full model: adjusting for all covariates. * p < 0.05, ** p < 0.01, *** p < 0.001

|  | **No change (ref.)** | **Gain** | **Both** | **Loss** |
| --- | --- | --- | --- | --- |
| Female |  | 0.304^*^ | 0.087 | 0.212 |
| 18-35 years (ref.) |  | 0.000 | 0.000 | 0.000 |
| 36-59 years |  | -0.921^***^ | -1.718^***^ | -0.483^*^ |
| 60 or older |  | -1.308^***^ | -2.615^***^ | -0.689^**^ |
| Migration Background |  | 0.561^*^ | 0.689^***^ | 0.470^**^ |
| Highest Educational Level: Compulsory (ref.) |  | 0.000 | 0.000 | 0.000 |
| Post-secondary |  | 0.001 | 0.307 | 0.178 |
| Tertiary |  | 0.463^*^ | 0.711^**^ | 0.543^**^ |
| Household Income: <900 Eur/month (ref.) |  | 0.000 | 0.000 | 0.000 |
| 900-1499 Eur |  | -0.068 | 0.165 | -0.256 |
| 1500-2599 Eur |  | -0.099 | -0.138 | -0.083 |
| 2600-3999 Eur |  | -0.429^*^ | -0.183 | -0.039 |
| 4000-5999 Eur |  | -0.245 | -0.186 | -0.141 |
| 6000 or more |  | -0.151 | 0.249 | -0.342 |
| Employment: Full-time (ref.) |  |  |  |  |
| Part-time |  | 0.178 | 0.607^**^ | 0.113 |
| Retired |  | 0.189 | 0.175 | 0.009 |
| Inactive |  | -0.225 | 0.857^***^ | 0.217 |
| Urban |  | -0.014 | 0.265 | 0.055 |
| East |  | 0.249^*^ | -0.145 | -0.175 |
| Constant |  | -1.278^***^ | -1.415^***^ | -0.565^*^ |

**Supplementary Table 3: Multinomial Logistic Regressions** Source: Source: Survey "Living in exceptional circumstances", Wave 3 (spring 2021). n= 3,713 respondents, 18-98 years. Multivariate multinomial logistic regression models, weighted, Relative Risk Ratios. Gain resp. loss of friends and acquaintances jointly modelled (1=gain / loss of either).

**Supplementary Figure 1: Bivariate Linkages between Socio-demographic and Socio-Economic Factor and Categorial Change variable** Source: Survey "Living in exceptional circumstances", Wave 3 (spring 2021). n= 3,713 respondents, 18-98 years. Weighted figures. Gain resp. loss of friends and acquaintances jointly modelled (1=gain / loss of either)
